# Navigating the Uncommon: PCI to an Anomalous Right Coronary Artery With a Malignant Course Following Failed Arterial Grafts

**DOI:** 10.1155/cric/8125980

**Published:** 2025-05-12

**Authors:** Ayman Helal, Mohsin Farooq, Mohamed Alama, Ibrahim Antoun

**Affiliations:** ^1^Department of Cardiology, Kettering General Hospital, University Hospitals of Northamptonshire (UHN), Kettering, UK; ^2^Cardiology imaging, Department of Cardiology, Kettering General Hospital, University Hospitals of Northamptonshire (UHN), Kettering, UK; ^3^Department of Cardiovascular Sciences, University of Leicester, Leicester, UK

## Abstract

A 67-year-old male presented with non-ST elevation myocardial infarction (NSTEMI) with underlying anomalous origin of the right coronary artery (RCA) and a malignant course between the aorta and pulmonary artery. Previous CABG with LIMA and RIMA grafts had failed, leaving the patient with severe in-stent restenosis (ISR) in the LAD and degenerated, blocked grafts. The patient underwent percutaneous coronary intervention (PCI) to the anomalous RCA, which posed challenges in engagement due to its anomalous course. This case report discusses the complexities of managing such anatomies, emphasizing the role of CT coronary angiography, the difficulty of engaging anomalous arteries, and the techniques used to achieve successful PCI.

## 1. Introduction

Anomalous coronary arteries are a rare but critical entity in interventional cardiology due to their potential for malignant courses and life-threatening complications, including myocardial ischemia and sudden cardiac death. When such anomalous vessels are involved in patients with previous coronary artery bypass grafting (CABG) failures, the complexities of revascularization significantly increase. This case report presents the challenges encountered in percutaneous intervention to an anomalous right coronary artery (RCA) originating from the left coronary cusp (LCC), which took a malignant course between the aorta and pulmonary artery. We also highlight the importance of multimodality imaging, particularly CT coronary angiography, in planning and guiding such interventions, along with a discussion on the approach to managing anomalous coronary arteries.

## 2. Case Presentation

**Figure 1:**
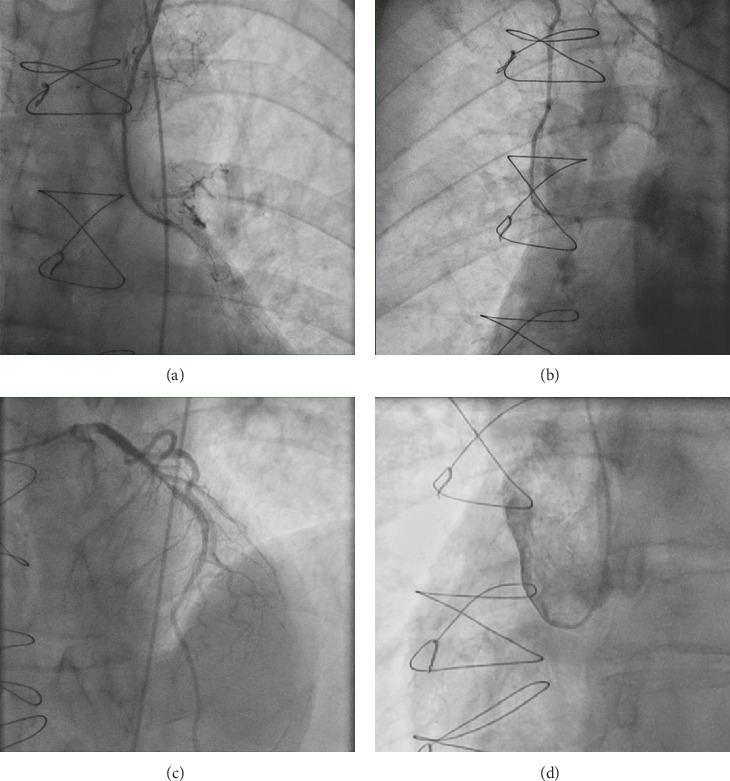
Diagnostic coronary angiogram showed complete occlusion of the LIMA-LAD (a) and RIMA-RCA (b) grafts, severe in-stent restenosis (ISR) of the mid-LAD stent (c), and failure to identify the native RCA ostium even with aortic root injection (d).

A 67-year-old male with a history of NSTEMI 12 years ago (treated by PCI top LAD) and previous CABG 11 years ago (LIMA to LAD and RIMA to RCA) presented with recurrent chest pain and dynamic T-wave inversions on the anterior leads. His troponin level was elevated (2476 ng/L), and he had been noncompliant with medication for 2 years. Coronary angiography revealed severe in-stent restenosis (ISR) of the mid-LAD stent and complete occlusion of the LIMA-LAD and RIMA-RCA grafts. Efforts to engage the native RCA were unsuccessful, and the procedure was halted.  Figure 1 demonstrated the diagnostic coronary angiogram findings.

**Figure 2:**
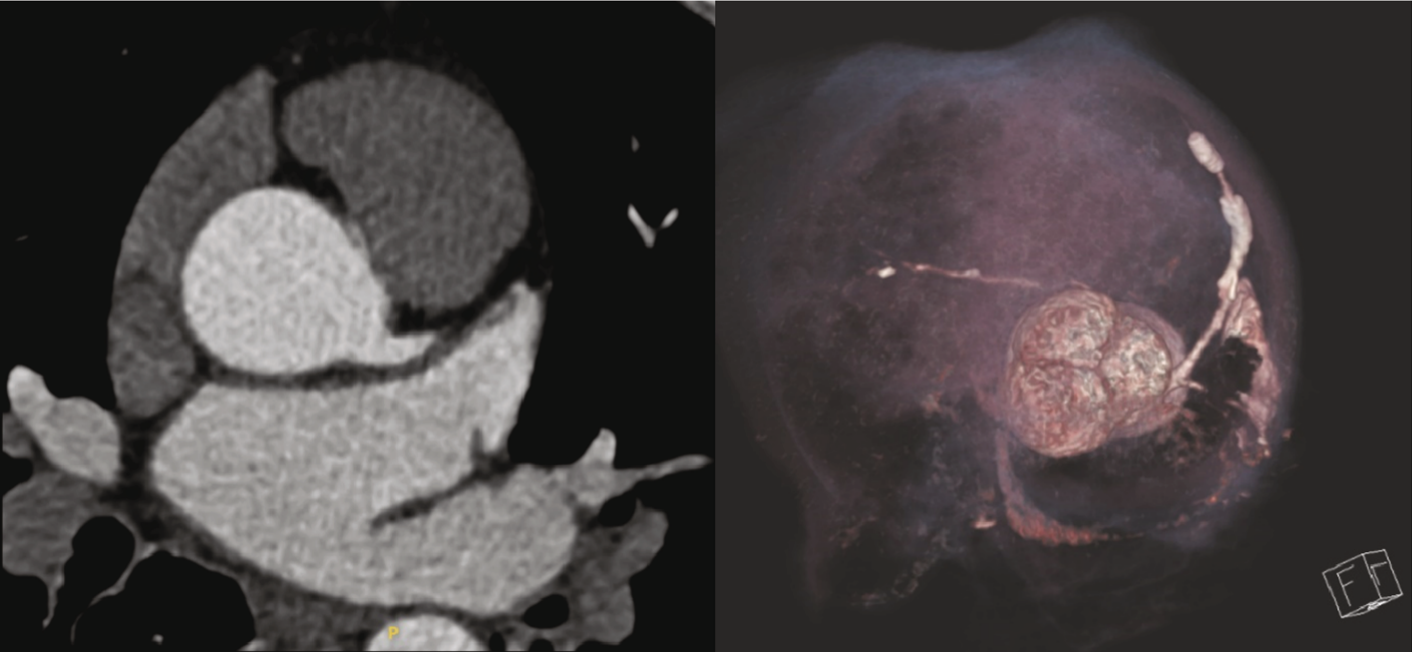
CT coronary angiogram showed anomalous origin of the RCA from the left coronary cusp, with a malignant course between the aorta and pulmonary artery.

CT coronary angiography was performed to further assess the anatomy, revealing an anomalous origin of the RCA from the LCC, with a malignant course between the aorta and pulmonary artery (Figure 2). The patient had severe left ventricular systolic dysfunction (EF 30%–35%) and akinesis of the anterior, lateral, and inferior walls and inferior septum on echocardiogram.

**Figure 3:**
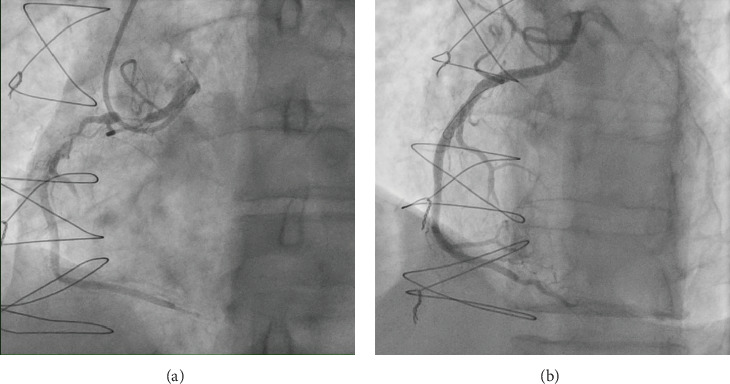
The anomalous RCA showed two significant subtotal occlusions in the proximal to mid segments (a) treated by two DES with good final angiographic result and TIMI-3 flow (b).

A repeat angiogram showed the RCA with two significant subtotal occlusions in the proximal to mid segments (Figure 3). Various guiding catheters, including AL 0.75, AL 1, AL 1.5, and MP1, were tried without successful engagement. Eventually, an EBU 3.75 6F catheter was able to engage the anomalous RCA. Wire advancement was challenging due to lack of support, so guide extension catheter (GuideLiner) was used to provide extra support. Lesion preparation was performed using escalating balloon diameters (Ryurei 1.25 × 15, 2.25 × 15, and then 3.0 × 20 were used). PCI was performed with two drug-eluting stents (DES): XIENCE Skypoint 3.5 × 48 mm to mid RCA, then a XIENCE PRO 4.0 × 23 proximally. Stent optimization was achieved using noncompliant NC TREK balloons (4.0 × 23 for the whole stent and a 4.5 × 15 to the mid and proximal segments). The final angiographic was good with TIMI-3 flow, and the patient was managed with dual antiplatelet therapy (DAPT) and heart failure medications with a plan for PCI to LAD ISR using laser atherectomy in another session.

## 3. Discussion

Anomalous coronary arteries are relatively rare, with an incidence of less than 1% in the general population. However, their clinical significance is profound, particularly when the coronary artery has a malignant course between the aorta and pulmonary artery. This anatomical variant can lead to extrinsic compression of the artery during physical exertion, contributing to ischemia and sudden cardiac events. An anomalous origin of the RCA from the left sinus is a very rare anomaly and accounts to 0.019%–0.49% of patients undergoing coronary angiography [1].

In this case, the anomalous origin of the RCA significantly complicated the procedure, as engagement of the RCA proved challenging due to its location and course. Multiple guiding catheters were trialed before achieving successful engagement with the EBU 3.75 6F catheter. This highlights the importance of catheter selection and operator experience in navigating anomalous coronary anatomies. Additionally, the use of a guide extension catheter (GuideLiner) was pivotal in providing adequate support for wire advancement and stent deployment.

CT coronary angiography played a crucial role in this case, as it allowed precise visualization of the anomalous RCA and its course, guiding the PCI strategy. Multimodality imaging, particularly in cases with complex anatomy or previous graft failures, is essential for planning revascularization [2, 3]. CT also aids in identifying the exact anatomical origin and course of anomalous vessels, which is critical in deciding the approach for PCI.

Several techniques were proposed to engage the ACAOS. In addition to using a guide extension catheter (GuideLiner) for enhanced support, modifying the distal tip of a JL-type guiding catheter to achieve a 90° rightward bend can provide a more coaxial alignment, improving stability during PCI. This technique has been documented in previous literature and may be particularly useful when conventional guide catheters fail to provide adequate backup support. The choice between these techniques should be tailored to the specific anatomical challenge and operator experience to optimize procedural success [4].

Anomalous coronary arteries originating from the opposite sinus of Valsalva (ACAOS) present a significant challenge due to their diverse anatomical and clinical manifestations. Although rare, the detection of ACAOS is increasing with the widespread use of noninvasive imaging for coronary artery disease [5]. However, in the absence of clear evidence-based guidelines, physicians face uncertainty in determining the best treatment approach. Surgical correction is not always justified based solely on the presence of ACAOS, making it crucial to conduct detailed anatomic and hemodynamic evaluation. Both invasive and noninvasive multimodality imaging play a key role in determining whether ACAOS is an incidental finding, the cause of symptoms, or a risk factor for sudden cardiac death. Recent data emphasize that myocardial ischemia in ACAOS is influenced by both fixed and dynamic factors, with varying anatomic high-risk features requiring individualized therapeutic decisions [6].

In cases where an ACAOS was suspected in the setting of acute myocardial infarction where prior coronary CT angiography may not be available, when difficulty is encountered in engaging the RCA with standard right coronary catheters, a practical approach is to perform a hand or aortic root contrast injection. This can provide an immediate clue to the anomalous origin by visualizing the contrast filling the artery from an unexpected location. Recognizing this anatomical variant early allows for a more efficient catheter selection strategy, reducing procedure time and contrast load while optimizing revascularization outcomes in emergent scenarios [7].

Interventions for anomalous coronary arteries remain controversial. Surgical revascularization, such as CABG, is often preferred, especially in cases with a malignant course. However, this patient had previously failed arterial grafts, necessitating a PCI approach. The success of PCI in such cases is highly dependent on the ability to engage the vessel, the availability of appropriate imaging modalities, and the expertise of the interventional cardiologist [8, 9].

## 4. Conclusion

This case demonstrates the complexities of managing a patient with an anomalous malignant RCA and failed arterial grafts. The difficulties encountered in engaging the anomalous artery and the role of CT coronary angiography in guiding PCI underscore the need for a tailored, patient-specific approach. Despite the challenges, successful revascularization was achieved with stents implantation, and the patient was discharged with a plan for continued medical management and PCI to LAD ISR using laser atherectomy in another session.

## Data Availability

Data from this study is available on request.

## References

[1] Lee B. Y. (2009). Anomalous Right Coronary Artery From the Left Coronary Sinus With an Interarterial Course: Is It Really Dangerous?. *Korean Circulation Journal*.

[2] Baz R. O., Refi D., Scheau C., Savulescu-Fiedler I., Baz R. A., Niscoveanu C. (2024). Coronary artery anomalies: A Computed Tomography Angiography Pictorial Review. *Journal of Clinical Medicine*.

[3] Helal A., Alama M., Hamid A., Nishtar S. (2024). Bioprosthetic Mitral Valve Thrombosis: The Role of Cardiac CT in Diagnosis and Guiding the Management. *BML Case Reports*.

[4] Lin C. C., Yeh K. H., Chou H. H., Hsu S. Y., Chang H. C. (2015). A Novel Technique for Percutaneous Coronary Intervention for Anomalous Right Coronary Artery Arising From the Left Sinus of Valsalva. *Acta Cardiologica Sinica*.

[5] Han P. L., Diao K. Y., Huang S. (2020). Anatomical Characteristics of Anomalous Left Coronary Artery From the Opposite Sinus (Left-ACAOS) and Its Clinical Relevance: A Serial Coronary CT Angiography Study. *IJC Heart & Vasculature*.

[6] Bigler M. R., Kadner A., Räber L. (2022). Therapeutic Management of Anomalous Coronary Arteries Originating From the Opposite Sinus of Valsalva: Current Evidence, Proposed Approach, and the Unknowing. *Journal of the American Heart Association*.

[7] Fretay X. H. D., Boudvillain O., Koutsoukis A., Degrell P., Dupouy P., Aubry P. (2025). Catheterization Techniques for Anomalous Aortic Origin of Coronary Arteries. *Catheterization and Cardiovascular Interventions*.

[8] Helal A., Cader A., Hetherington S., Hogrefe K. (2025). First Application of Combined Cutting Balloon and Rotational Atherectomy and Intravascular Lithotripsy in Old Degenerated Saphenous Venous Graft. *Catheterization and Cardiovascular Interventions*.

[9] Reul R. M., Cooley D. A., Hallman G. L., Reul G. J. (2002). Surgical Treatment of Coronary Artery Anomalies: Report of a 37 1/2-Year Experience at the Texas Heart Institute. *Texas Heart Institute Journal*.

